# Predictive Symptoms and Signs of Laboratory-confirmed Influenza: A Prospective Surveillance Study of Two Metropolitan Areas in Taiwan

**DOI:** 10.1097/MD.0000000000001952

**Published:** 2015-11-06

**Authors:** Jeng-How Yang, Po-Yen Huang, Shian-Sen Shie, Shuan Yang, Kuo-Chien Tsao, Tsu-Lan Wu, Hsieh-Shong Leu, Ching-Tai Huang

**Affiliations:** From the Division of Infectious Diseases, Department of Medicine (J-HY, P-YH, S-SS, H-SL, C-TH); and Department of Laboratory Medicine, Chang Gung Memorial Hospital and Chang Gung University, Taoyuan, Taiwan (YS, K-CT, T-LW).

## Abstract

Influenza infection poses annual threats and leads to significant morbidity and mortality. Early diagnosis is the key to successful treatment. Laboratory-based diagnosis has various limitations. Diagnosis based on symptoms or signs is still indispensable in clinical practice. We investigated the symptoms or signs associated with laboratory-confirmed influenza.

A prospective study across 2 influenza seasons was performed from June 2010 to June 2012 at 2 branches (Taipei and Lin-Kou) of Chang Gung Memorial Hospital. Patients who visited outpatient clinics with suspected acute respiratory tract infection were sampled by throat swab or nasopharyngeal swab. RT-PCR and/or virus culture were used as a reference standard. We used logistic regression to identify the symptoms or signs associated with laboratory-confirmed influenza infection. We also evaluated the performance metrics of different influenza-like illness used in Taiwan, the USA, and WHO.

A total of 158 patients were included in the study. The prevalence of influenza infection was 45% (71/158). Fever, cough, rhinorrhea, sneezing, and nasal congestion were significant predictors for influenza infection. Whereas fever + cough had a best sensitivity (86%; confidence interval [CI] 76%–93%), fever + cough and sneezing had a best specificity (77%; CI 62%–88%). Different case definitions of influenza-like illness had comparable accuracy in sensitivity and specificity.

Clinical diagnosis based on symptoms and signs is useful for allocating resources, identifying those who may benefit from early antiviral therapy and providing valuable information for surveillance purpose.

## INTRODUCTION

In April 2009, a novel influenza virus, influenza A (H1N1)pdm09, appeared in Mexico and subsequently spread worldwide quickly within several months, leading to considerable death toll among healthy adults.^1^ Certain genetic and virology characteristics of the virus revealed a close relationship with swine-origin influenza viruses.^2^ Lack of immunity among most human populations and the high fatality rates by animal studies raise the concerns that the novel virus will mimic the 1918 “Spanish” influenza and further cause millions of deaths.^3^ To mitigate against the spreading of the virus, the government has applied various measures such as antiviral agent treatment, traffic control bundles, as well as a mass vaccination program.^4–6^

Whereas the epidemiological data over 2009/2010 pandemic season suggested that influenza A (H1N1)pdm09 shared a similar mortality rate with circulating influenza viruses,^7^ other studies have demonstrated greater burdens and higher proportions of severe illness in the subsequent postpandemic seasons.^8,9^ In the USA, influenza infections account for 3 to 5 million illnesses and 500,000 deaths each year.^10^ By constant alteration of genetic information, the revolutionary change of influenza A viruses across different hosts or species may cause another pandemic and pose a threat to our healthcare systems.^11^ We are still under the shadow of this possibility.

Then establishing a reliable diagnosis of influenza becomes increasingly important. A number of diagnostic tools have developed, but all are with limitations. Rapid influenza diagnostic test (RIDT) produces readily available results, but the poor sensitivity makes it unsuitable for screening in clinical settings.^12,13^ Molecular diagnosis by RT-PCR is more sensitive, but generally more expensive than the other tests. It also requires technical support and the result is not always available onsite to change the clinical decisions.^14^ Virus isolation by conventional culture takes about 1 week and is not practical for diagnosis, which needs prompt management, such as cohorting, physical distancing, or pharmaceutical interventions. Diagnosis based on symptoms or signs is indispensable in clinical practice.

Symptoms of influenza infection include fever, cough, sore throat, sneezing, rhinorrhea, nasal congestion, headache, malaise, myalgia, nausea, vomiting, and diarrhea. None of them is specific and the symptoms can be caused by numerous respiratory viruses.^15^ People have claimed that “influenza-like illness” (ILI)—cold symptoms with severer systemic manifestations such as sudden onset of high fever, myalgia, and protracted malaise—is more likely to be caused by influenza virus infection. Many studies have evaluated the performance of different symptoms or case definitions of ILI. Application and interpretation of the findings of these studies are hampered by differing methodologies, varying clinical settings, different inclusion criteria, and inconsistent conclusion.^16,17^ One meta-analysis, analyzing 915 studies, has concluded that few studies complied with the standard as prospective design, clinical symptom assessment, as well as laboratory-confirmed cases.^18^

Primary care physicians still rely on symptoms to make a clinical diagnosis. Symptomatic predictors for identifying influenza infection are essential for rapid intervention, timely antiviral therapy, and isolation of patients in outbreak settings. Defining a reliable criterion with appropriate sensitivity and specificity to distinguish influenza from other respiratory infection is imperative. Our aim of the study is to determine the most predictive symptoms of influenza infection and to evaluate the diagnostic performance of different case definitions of ILI with a reference standard.

## METHODS

We conducted a prospective surveillance at outpatient services of Chang Gung Memorial Hospital Linkou and Taipei Branch, which proved both primary and tertiary care in 2 metropolitan areas (Taoyuan and Taipei) of northern Taiwan, from June 2010 to February 2012. Throat swab or nasal swab was performed by physicians in adult patients (>18 years old) who presented with upper respiratory tract symptoms, defined as one of the following: fever, cough, chills, headache, malaise, sore throat, rhinorrhea, nasal congestion, or myalgia. Specimens from upper respiratory tracts were sent to the laboratory medicine department for RT-PCR and/or virus culture as a part of the daily procedure. RIDT could be performed by trained physicians at clinical discretion. A questionnaire was completed with the assistance of the trained assistant immediately. Demographics, underlying conditions, vaccination status, cluster, date of fever onset, and relevant symptoms were recorded. Written informed consent was acquired for each patient. This study was approved by Chang Gung Memorial Hospital Institutional Review Board (99–0786B).

The laboratory tests were performed after the procedures described previously.^19^ Briefly, the specimens for both RT-PCR and virus culture were stored at 4°C in a virus transport medium and sent to the virology laboratory within 30 minutes of being sampled. The specimens were inoculated with standardized cell lines, and cytopathic effect was checked on a daily basis. Viral RNA was extracted by MagNA PURE Autoextractor with MagNA Pure LC Total Nucleic Isolation Kit (Roche Diagnostics, Germany). The specimens for RIDT were sent to the laboratory within 30 minutes of being sampled and processed in the laboratory by trained technicians under the manufacturer's instructions (QuickVue, influenza A + B). Patient specimens were placed in a reagent tube for virus nucleoprotein extraction. A test strip containing mouse monoclonal anti-influenza virus A and B antibodies was placed in the reagent tube for detection of virus antigens. Virus culture was transported by a virus isolation transport medium.

Statistical analyses were performed using the R software/environment.^20^ Continuous variables were compared using Student *t* test or Mann-Whitney *U* test. Binomial variables were compared using chi-square or Fisher exact test. For multivariate analysis, a binary logistic regression model was constructed with selected variables by stepwise procedures. A 2-tailed *P* value <0.05 was considered statistically significant in all tests. The diagnostic accuracy of significant symptoms in multivariate analysis and their combinations were determined by sensitivity, specificity, positive predictive value (PPV), negative predictive value (NPV), positive likelihood ratio (PLR), and negative likelihood ratio (NLR). Similarly, performance metrics of ILI case definitions of the USA,^21^ Taiwan,^22^ and WHO^23^ were evaluated, respectively. All performance parameters were determined against RT-PCR and/or viral culture as a reference standard. As a part of their daily work, 3 different divisions of the laboratory medicine department performed RT-PCR, viral culture, and RIDT, respectively. Therefore, the readers of the reference standard (RT-PCR and/or viral culture) were blind (masked) to the reader of the other tests and unaware of the clinical symptoms of the patients.

## RESULTS

**FIGURE 1:**
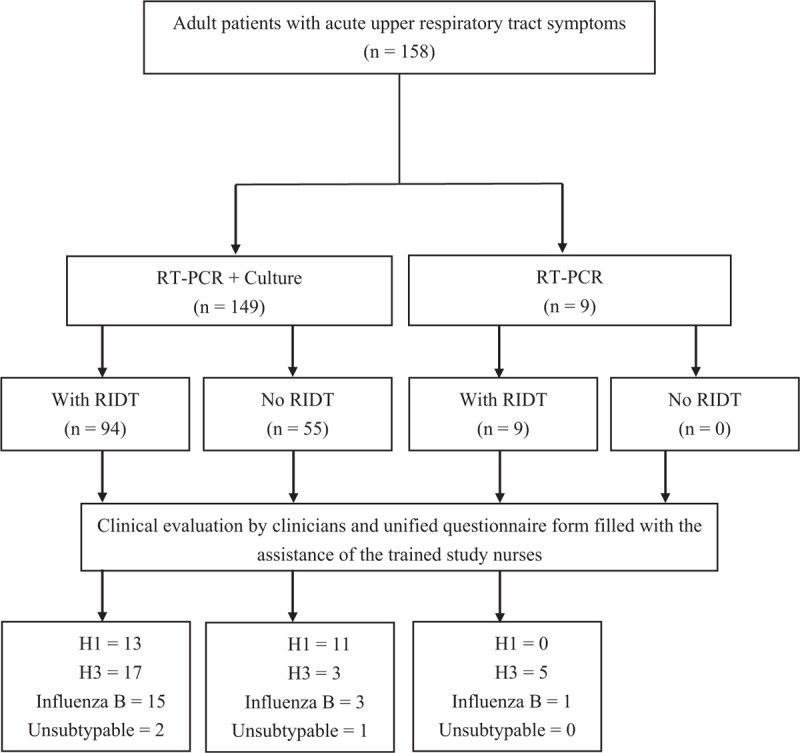
Flow diagram of the patients underwent standardized diagnostic tests (RT-PCR with or without virus culture) and rapid influenza diagnostic test (RIDT). H1 = influenza A(H1N1)pdm09; H3 = influenza A(H3).

During the study period, the study included a total of 158 patients with 158 specimens tested for RT-PCR, 149 for virus culture, and 103 for RIDT (Fig. 1). About half of the specimens were tested positive for influenza virus infection by RT-PCR or viral culture, and the prevalence of influenza infection was 45% (71/158). The identified viruses were influenza A (H3) (n = 25), followed by influenza A (H1N1)pdm09 (n = 24), influenza B (n = 19), and unsubtypable influenza A virus (n = 3). Among one-third of the patients who received RIDT at clinicians’ discretion, half of them were positive for influenza infection (49%, 50/103) and most were subsequently confirmed by RT-PCR or virus culture (80%, 40/50). The false-negative rate of RIDT was 26% (13/53) (Table 1).

**TABLE 1:**
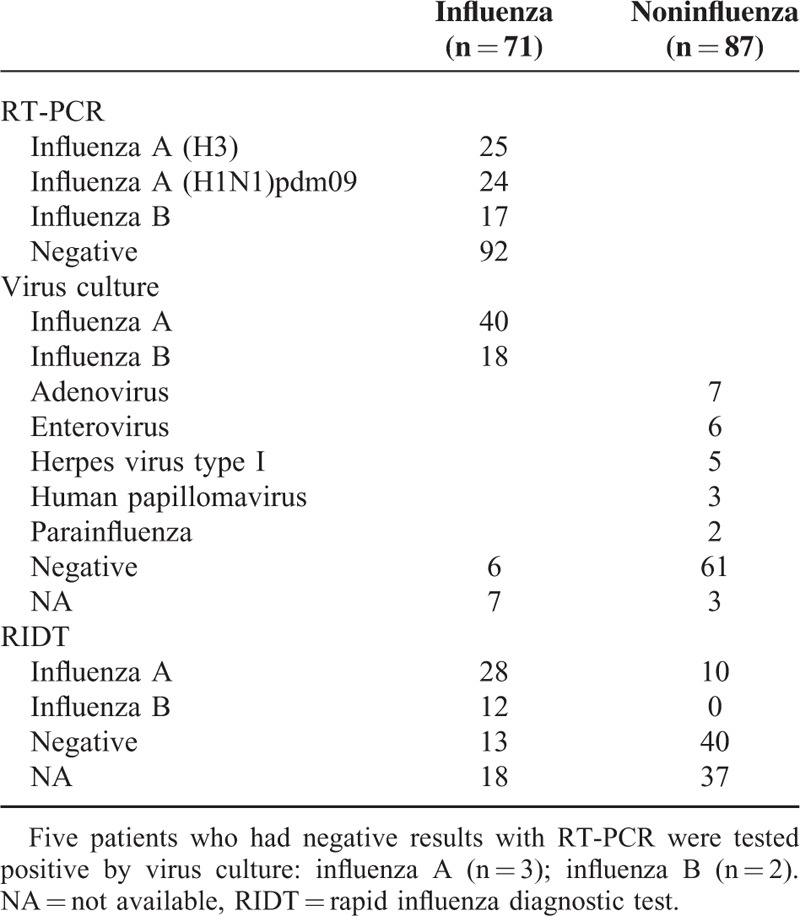
Laboratory Results of 158 Patients With Acute Upper Respiratory Tract Symptoms

Demography data, prior vaccination status, and symptoms and signs of 71 laboratory-confirmed influenza patients were compared with the other 87 patients who were tested negative for influenza infection (Table 2). Few patients had comorbid illnesses. In univariate analysis, patients with influenza infection were more likely to have a fever (odds ratio [OR] 4.14, 95% confidence interval [CI] 1.76–9.78, *P* < 0.001), cough (OR 13.14, 95% CI 2.98–57.87, *P* < 0.001), rhinorrhea (OR 2.58, 95% CI 1.29–5.14, *P* = 0.006), nasal congestion (OR 2.77, 95% CI 1.42–5.32, *P* = 0.002), and sneezing (OR 3.04, 95% CI 1.58–5.84, *P* < 0.001). Vaccination apparently was a protective factor for influenza infection, but it was not statistically significant (OR 0.54, 95% CI 0.26–1.10, *P* = 0.089). There was no difference in age, sex, onset of symptoms, and history of cluster in family or colleagues. In multivariate analysis, the most predictive symptoms of influenza infection were fever (OR 5.43, 95% CI 2.19–14.83, *P* < 0.001), cough (OR 16.72, 95% CI 4.37–110.73), and sneezing (OR 2.69, 95% CI 1.25–5.94). Although not statistically significant, the days from the onset of symptoms to the date of hospital visit were shorter in patients with influenza (OR 0.89, 95% CI 0.78–0.99, *P* = 0.055). Vaccination might protect people from getting influenza infection, but this was not statistically significant (OR 0.51, 95% CI 0.22–1.19, *P* = 0.123).

**TABLE 2:**
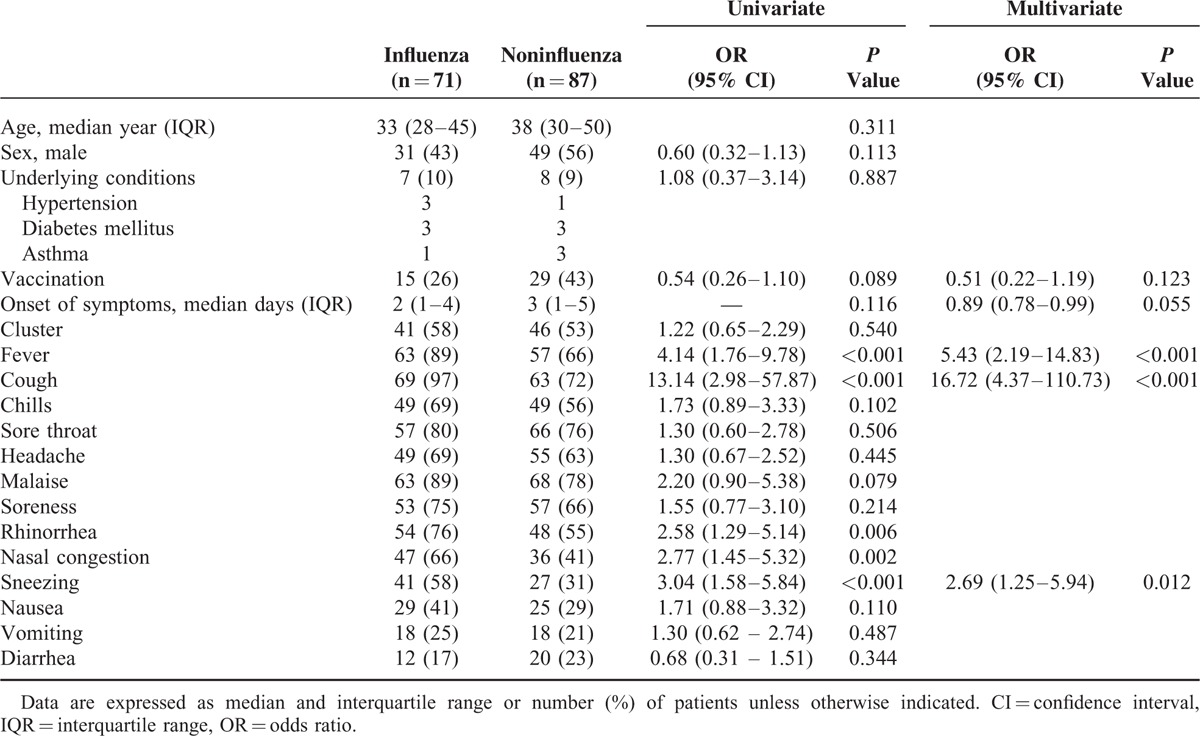
Univariate Analysis of Symptoms and Signs for Patients With or Without Laboratory-confirmed Influenza Infection

Among the combinations of the significant symptoms by multivariate analysis, fever + cough were highly sensitive, but not so specific (sensitivity = 0.86, 95% CI 0.76–0.93; specificity = 0.58, 95% CI 0.46–0.68). Fever + cough and sneezing was not sensitive, but highly specific (sensitivity = 0.50, 95% CI 0.39–0.63; specificity = 0.87, 95% CI 0.79–0.94). RIDT performed well in our study (sensitivity = 0.75, 95% CI 0.62–0.86; specificity = 0.80, 95% CI 0.66–0.90). Whereas the case definitions of ILI had high sensitivity and modest specificity, different definitions of ILI + RIDT were highly specific (0.93–0.97) (Table 3).

**TABLE 3:**
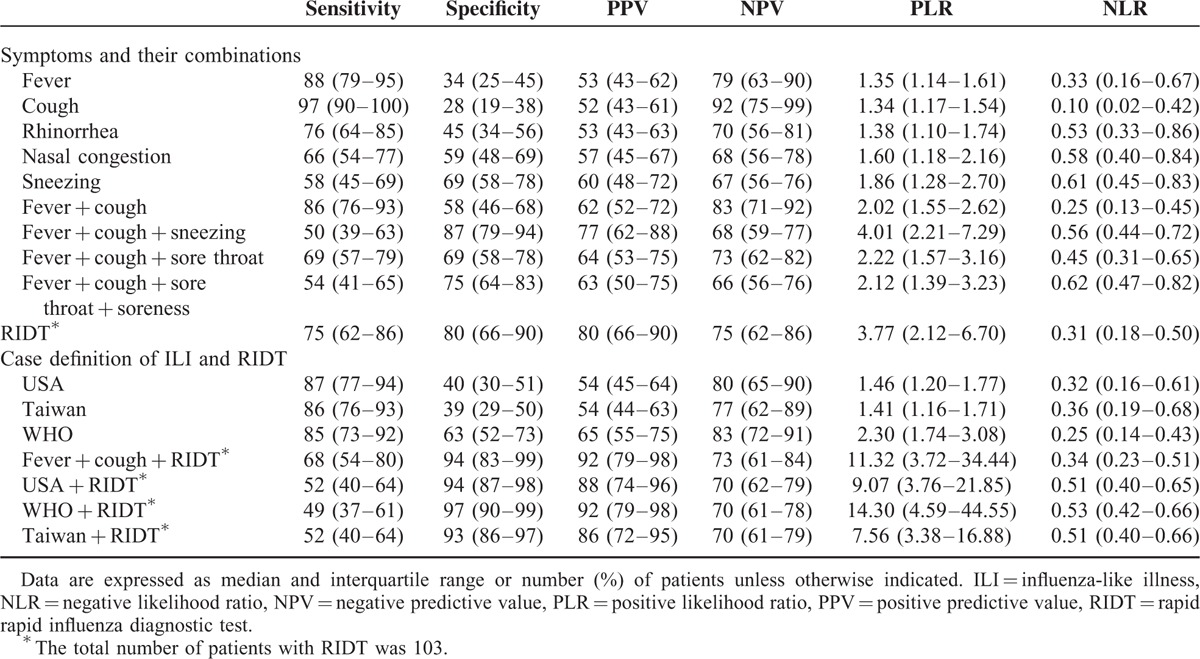
Diagnostic Accuracy of Symptom + Their Combinations, Rapid Influenza Diagnostic Test (RIDT), and Case Definition of Influenza-like Illness (ILI)

## DISCUSSION

Diagnosing influenza infection in primary care settings is a challenge as similar symptoms can occur in the patients infected with other respiratory viruses. Laboratory tests such as RIDT, virus culture, or RT-PCR have various limitations. In this study, we investigated the most predictive symptoms associated with laboratory-confirmed influenza and compared the performance between different case definitions of ILI. Our results show that in patients who visit the outpatient clinics, fever + cough has high sensitivity and increases the likelihood of influenza. Fever + cough and sneezing have high specificity to rule in the diagnosis of influenza. We also showed that case definitions of ILI used in the USA, Taiwan, and WHO have comparable performance and are useful for screening due to their high sensitivity and modest specificity.

Although some symptoms such as fever or cough alone had good sensitivity and high negative PPV, no single symptoms yielded a summary PLR greater than 2 in the study (Table 3). This finding is in agreement with those of a large meta-analysis, and suggests that clinicians cannot rule in influenza infection according to single symptom.^18^ On the contrary, the diagnostic performance is more encouraging for the combinations of symptoms. Whereas fever + cough had best sensitivity and good NPV, fever + cough and sneezing had best specificity and PPV in our analysis. Both criteria have moderate PLR and NLR. On the basis of these findings, it is reasonable to consider the usefulness of fever + cough and sneezing to diagnose influenza infection. This also accords with those of prior studies and supports the use of certain symptoms to rule in or rule out the diagnosis of influenza.^15–18,24–27^

Contrary to our results, Govaert et al^28^ have demonstrated a high specificity (0.95), yet a low sensitivity (0.27), among patients aged 60 or older. They investigated the predictive value of symptomology in a large group of patients (n = 1838) and used serologically confirmed influenza as a reference standard. They concluded that the combinations of fever, cough, and acute onset were useful to predict influenza infection. The PPV (0.30) and sensitivity (0.27) of their study were much lower than those of the other studies. This may be partly related to the low prevalence of influenza infection (6.6%, 121/1838). Moreover, some evidence suggests that the estimated sensitivity and specificity of a diagnostic test may vary with the disease prevalence of interest.^29^ Another possible issue in their study is selection bias, as the study assesses all the persons enrolling in the vaccine trial, whether they had respiratory symptoms or not. Despite these limitations, the results of Govaert et al still support the use of certain symptoms to predict influenza infection.

In our analysis, results with the case definitions of ILI used in the USA, Taiwan, and WHO have high sensitivity (0.85–0.87) and modest specificity (0.39–0.63). When compared with one another, the WHO criterion has the highest specificity (0.63), as well as PLR (2.30), indicating their role in ruling in influenza infection and changing a clinician's judgment in 1 patient.^30^ Our data confirm and are in accordance with those of a recent study, which also favors the use of the WHO criterion.^25^ This differs from that of a study conducted by Chen et al^31^ in Taiwan. They demonstrated low sensitivity (0.58) and high specificity (0.74) by using ILI definition of Taiwan. Whereas we used a standardized reference standard (RT-PCR and/or virus culture) to diagnose influenza infection, they enrolled the patients with positive RIDT with or without virus culture. It may result in an overestimated sensitivity due to insensitive RIDT, as well as lower prevalence (36%, 370/815), in their study.

We have previously demonstrated that when the activity of influenza virus in a community is low, fever + cough + chills, with clustering of cases in the workplace or household, has the highest likelihood of influenza A infection.^32^ The results of this study are different from that of our prior study with respect to significant symptoms-associated influenza infection because of the low prevalence of influenza infection at that time (45% vs 5%). It implies that cluster may be not significant in influenza season since the virus could infect 1 person more easily when the activity of influenza virus is high in a community.

Interestingly, our study shows a good performance of RIDT (Table 3). Although it is useful for confirming the diagnosis with its high specificity and short turnaround time, the performance of RIDT may be influenced by patient age, duration of illness, and virus subtypes, and perhaps disease severity.^12,19,33–36^ Our patient populations are relatively younger and less ill than those of the other studies, probably leading to a better sensitivity with RIDT. Yet, we do not assess if these patients have higher viral loads in their upper respiratory tracts. Further study is needed to evaluate this possibility.

There are potential limitations to this study. The validity of this single-center study may be compromised in selecting cases, gathering information, collecting specimens, and making comparisons. First, our study population is comprised of individuals who have uncomplicated influenza and few underlying diseases. The severity of illness was not evaluated and apparently easy conditions were noted in the studied patients. The results may not be applicable in the patients with severe illness and admission due to influenza infection. However, few studies explore the association between symptoms and laboratory-confirmed influenza in a prospective manner. With this surveillance across 2 influenza seasons in Northern Taiwan, valuable insight is provided for the primary care physicians. Diagnosis can be made on clinical grounds and intervention measures like antiviral therapy may be initiated earlier in selected cases to further reduce medical costs and even mortality.^37^ Second, patients included in this study were selected and sampled by the physicians in the emergency department and outpatient clinic of the infectious disease division as part of the daily routine. Despite a standardized form record and unified protocols for specimen processing were used, interobserver variations may exist and bias the estimates of the study. Some patients who present with similar symptoms may not go with the same laboratory test as those without symptoms. Nevertheless, many of the patients presented with ILI to our hospital would visit emergency department or outpatient clinic of the infectious disease division. Therefore, we presume that this limitation may affect each group of the patients and may not alter the main results of our study. Although we demonstrate a trend of the protective effect of vaccination against influenza infection, it is not statistically significant partly due to the scanty case number and older age of the patients (>18 years). Finally, we did not investigate different symptoms occurring in various subtypes of influenza infection because of a limited number of cases. Further large multicenter studies would be required to elucidate this issue.

## CONCLUSIONS

In conclusion, our results reinforce the importance of predictive symptoms and signs for the diagnosis of influenza infection in clinical settings. In particular, fever + cough with or without sneezing is useful for the purpose of screening, as well as surveillance. Since every laboratory tests have limitations, it is suggested that clinicians perform appropriate tests among selected cases with the use of clinical symptoms or different case definitions of ILI. Effective allocation of limited resources like antiviral therapy, as well intervention measures, can also be achieved with appropriate diagnosis of influenza infection in clinical settings.
